# Dynamic Calibration of Quartz Flexure Accelerometers

**DOI:** 10.3390/s25165096

**Published:** 2025-08-16

**Authors:** Xuan Sheng, Xizhe Wang, Wenying Chen, Yang Shu, Kai Zhang

**Affiliations:** Institute of Systems Engineering, China Academy of Engineering Physics, Mianyang 621999, China; shengxuan23@gscaep.ac.cn (X.S.); wangxizhe23@gscaep.ac.cn (X.W.); mysysysy@163.com (Y.S.)

**Keywords:** dynamic behavior, quartz flexure accelerometers, calibration method, dual-axis precision centrifuge

## Abstract

**Highlights:**

**What are the main findings?**

- A dynamic calibration model is developed based on a mechanistic analysis of quartz flexure accelerometers.
- A dynamic calibration method is designed utilizing a dual-axis precision centrifuge in conjunction with the dynamic model.

**What is the implication of the main finding?**

- The magnitude of dynamic measurement errors is significantly reduced by the proposed dynamic error model.
- The calibration of the dynamic parameter yields a relative standard deviation of −0.048%.

**Abstract:**

The dynamic behavior of quartz flexure accelerometers remains a subject of ongoing investigation, particularly in areas such as theoretical modeling, standardization, calibration methodology, and performance evaluation. To address the limitation of conventional static calibration models in accurately representing accelerometer responses under dynamic acceleration excitation, a dynamic calibration model is proposed. A mathematical model is first developed based on the physical mechanism of the accelerometer, characterizing its intrinsic dynamic response. Simulation-based analysis demonstrates that the proposed dynamic model offers significantly improved accuracy compared to traditional static approaches. Furthermore, a dynamic calibration method leveraging a dual-axis precision centrifuge is designed and validated. The results confirm that the proposed approach enables the precise calibration of quartz flexure accelerometers in accordance with the dynamic model. The calibration of the dynamic parameter yields a relative standard deviation of −0.048%.

## 1. Introduction

Accelerometers, as core sensing elements, have been widely employed in critical fields such as inertial navigation, attitude control, and the monitoring of ships and vehicles [1,2]. With the continuous advancement of related technologies, the demand for accelerometers with higher accuracy has increased substantially [3,4,5]. Owing to their pivotal role in vibration measurement and navigation control systems, the precision of accelerometers directly affects the overall accuracy and reliability of such systems [6]. Consequently, research into accelerometer error calibration and compensation has attracted sustained academic attention. In standard practices adopted in the United States and Europe, accelerometer error models are typically expressed as follows [7]:

(1) $$\begin{array}{cc} a_{s} & =\frac{E}{K_{1}}=K_{0}+a_{i}+K_{2}\;a_{i}^{2}+K_{3}\;a_{i}^{3}+\\ & \;K_{\mathrm{ip}}\;a_{i}\;a_{p}+K_{\mathrm{io}}\;a_{i}\;a_{o}+K_{\mathrm{op}}\;a_{o}\;a_{p}+\\ & \;\delta_{o}\;a_{p}-\delta_{p}\;a_{o}+\ldots \end{array}$$

where:

E is the sensor output (output unit);
$a_{s}$ is the indicated sensor output (m/s^2^);
$K_{0}$ is the bias (m/s^2^);
$K_{1}$ is the scale factor [output units/(m/s^2^)];
$K_{2}$ is the second-order coefficient [(m/s^2^)/(m/s^2^)^2^];
$K_{3}$ is the third-order coefficient [(m/s^2^)/(m/s^2^)];
$K_{\mathrm{ip}},K_{\mathrm{op}},K_{\mathrm{io}}$ are the cross-coupling coefficients [(m/s^2^)/(m/s^2^)];
$a_{i}$ is the acceleration along sensor IA (m/s^2^);
$a_{p}$ is the acceleration along sensor pendulous axis (PA) (m/s^2^);
$a_{o}$ is the acceleration along sensor output axis (OA) (m/s^2^);
$\delta_{o}$ is the misalignment angle between IA and case reference IA about OA (rad);
$\delta_{p}$ is the misalignment angle between IA and case reference IA about PA (rad).

Early research on accelerometer accuracy primarily focused on static errors, which are mainly caused by environmental factors such as temperature and are typically mitigated through calibration techniques [8,9,10,11,12]. Numerous advanced mathematical models have been developed to compensate for temperature-induced drift and to suppress static errors [13,14,15].

In most existing studies, especially in the fields of guidance and control, accelerometers are generally characterized by static models [16,17,18]. However, with the rapid advancement of aerospace and guidance technologies, accelerometers are now being deployed in increasingly complex and dynamic environments. In contrast to traditional applications, modern accelerometers are expected to accurately capture and process dynamic signals with high fidelity. Nevertheless, owing to their inherent dynamic characteristics, accelerometers often show discrepancies between the measured output and the true acceleration input, especially under high-frequency or rapidly varying excitations. Such discrepancies manifest as dynamic measurement errors, underscoring the necessity of developing dynamic models capable of accurately capturing the true behavior of accelerometers under real-world conditions.

For dynamic performance evaluation and model identification, international standards such as ISO 16063-32:2016 classify dynamic calibration into vibration and shock calibration [19]. These techniques apply known, traceable dynamic excitations (vibration or shock) and record the sensor’s response to develop its mathematical model [20].

Recent studies have proposed various methods for evaluating dynamic performance and calibrating dynamic parameters. Wang et al. [1] introduced an approach that employs multiple indices in both the time and frequency domains to evaluate dynamic performance, incorporating novel performance metrics. These indicators cover the time, frequency, and uncertainty domains, offering a comprehensive framework for assessing dynamic models. Geist et al. [21] investigated Type A and Type B uncertainties associated with triaxial accelerometer calibration, providing a statistical foundation for assessing model parameter accuracy. However, these studies did not propose concrete calibration methods.

In 2023, Zhang et al. [22] proposed a pulse-based dynamic calibration method using the Down-Step Response Method (DSRM) to measure frequency responses. This method utilized foam aluminum to generate shock signals and extracted time domain features such as overshoot and settling time to infer frequency characteristics. However, the method is hindered by high complexity and limited precision, rendering it unsuitable for high-accuracy applications. Wei et al. [23] introduced a dynamic model parameter identification approach based on discrete spectrum correction and least squares (DSC-LS), which effectively mitigates spectral leakage and achieves high identification accuracy. Nevertheless, the mathematical models of accelerometer dynamics often involve high-order differential equations, posing practical implementation challenges. Xiao et al. [24] proposed a method that integrates least squares estimation with adaptive neural networks to estimate dynamic linear components. Despite its theoretical potential, the method relies entirely on data and neglects the physical mechanism of the accelerometer, raising concerns about reproducibility and generalization.

Overall, current dynamic calibration methods still face significant limitations in both modeling and application. Many are difficult to implement in fields such as guidance and control, which demand extremely high standards for model accuracy, responsiveness, and real-time performance. In addition, the complexity of the models and calibration procedures often leads to increased cost, further limiting their feasibility in engineering practice.

One important reason for the limitations of existing calibration methods lies in the constraints imposed by the excitation equipment used to generate input accelerations. In practice, the performance and flexibility of the calibration device significantly affect the effectiveness, accuracy, and applicability of dynamic calibration techniques. Commonly used devices for accelerometer calibration include precision turntables (gravity-based), Hopkinson bars, precision vibration tables, and precision centrifuges.

Traditional static accurate calibration methods typically utilize precision turntables under Earth’s gravitational field [25,26,27]. By utilizing the Kalman filters, some nonlinear errors and dynamic performances of low-precision accelerometers can also be tested [28,29,30]. A key limitation of gravity-based calibration is its narrow input acceleration range (−1 g to +1 g), which restricts its applicability to high-acceleration scenarios. Shock calibration using Hopkinson bars offers a wider acceleration range but suffers from relatively low accuracy and poor repeatability [31].

Precision vibration tables can generate harmonic dynamic inputs, but their calibration uncertainty (typically on the order of $10^{-3}$) is significantly higher than that of precision centrifuges (typically on the order of $10^{-5}$) [32]. Moreover, due to their inherent mechanical limitations, the amplitude of acceleration generated by vibration tables depends on frequency and cannot be independently adjusted.

The precision centrifuge serves as a key inertial navigation testing apparatus for calibrating accelerometers under high-overload conditions [33,34]. The accuracy of the generated acceleration field directly determines the calibration precision of accelerometers under test. Precision centrifuges currently play a critical role in calibrating inertial instruments, particularly for accelerometers [35]. As a result, centrifuge-based calibration techniques have attracted growing attention in recent years. Qiao et al. [36] proposed a theoretical method for estimating nonlinear error coefficients of Pendulous Integrating Gyro Accelerometers (PIGAs) using a centrifuge. Sohrabi et al. [37] investigated the calibration of linear accelerometers using multi-position and multi-acceleration modes on a disk centrifuge. Sun et al. [38] derived a precise expression for the input acceleration to improve calibration accuracy. More recently, Sun et al. [39] developed a revised error calibration model for linear accelerometers on disk centrifuges, compensating for misalignment angles and installation errors.

However, these efforts primarily focused on static or quasi-static calibration, and their setups remain based on conventional single-axis disk centrifuges.

Traditional disk-type centrifuges can only generate static acceleration excitations and are unable to calibrate the dynamic characteristics of accelerometers. To overcome this limitation, dual-axis precision centrifuges, equipped with a secondary rotating axis mounted atop the primary one, have been developed to generate high-precision harmonic excitation signals [40].

Compared to other calibration devices, dual-axis precision centrifuges exhibit the following unique advantages:

1. Compared to precision vibration tables, they offer higher accuracy and independent control of excitation amplitude and frequency.
2. Compared to precision turntables, they enable a wider adjustable acceleration range, allowing for enhanced calibration precision.
3. Compared to Hopkinson bars, they provide better repeatability and consistency due to their mechanical stability and programmable control.

These capabilities render dual-axis precision centrifuges an optimal solution for dynamic calibration tasks requiring both precision and adaptability. However, there are currently few studies on the calibration of accelerometers using dual-axis precision centrifuges. Specifically, only a small number of studies have focused on high-precision, rapid static calibration of PIGA accelerometers via such centrifuges, while there remains a lack of research on calibrating the dynamic characteristics of accelerometers utilizing these devices.

In summary, although static calibration and compensation techniques for accelerometers are relatively mature, the increasing demand for capturing highly dynamic phenomena underscores the urgency of advancing internal dynamic performance evaluation and calibration techniques. Current research on dynamic calibration focused on sensor response characteristics still faces limitations in terms of dynamic mechanism modeling, calibration methods, and high-precision platforms. This paper aims to address the precise characterization and calibration of dynamic errors arising from the intrinsic dynamic response of high-accuracy accelerometers. By analyzing the internal mechanisms of quartz flexure accelerometers, we establish a hybrid error model—structurally simpler than existing dynamic models—that incorporates the conventional static model and newly introduced dynamic compensation terms. The proposed model demonstrates good agreement with the actual output of the accelerometer under low-order polynomial inputs and low-to-medium frequency sinusoidal excitations. Furthermore, a novel calibration method is developed based on a dual-axis precision centrifuge, enabling the simultaneous and accurate identification of both static and dynamic parameters, thereby achieving more comprehensive and precise accelerometer calibration.

The rest of this paper is structured as follows. Section 2 presents the structural and dynamic modeling aspects of quartz flexure accelerometers. Section 3 defines error metrics for dynamic input conditions and further analyzes the output of accelerometer and the calibration effectiveness of both static and dynamic models. Section 4 develops a calibration method for the dynamic model based on a dual-axis precision centrifuge. Section 5 analyzes the performance of the proposed method via simulation results. Finally, Section 6 concludes the paper with a brief summary.

## 2. Accelerometer Error Model Incorporating Dynamic Terms

Accelerometers come in many types, including pendulum-integrating gyroscope accelerometers, flexural pendulum accelerometers, and vibrating-wire accelerometers [13,18,41]. Among these, the quartz flexure accelerometer (hereinafter referred to as “the accelerometer”) has been widely employed in various fields due to its compact size, high precision, low power consumption, and excellent long-term stability [42,43].

This section presents a mechanism-based model grounded in the operational principles of quartz flexure accelerometers. By analyzing the accelerometer’s non-zero initial response to a basic dynamic input (i.e., a ramp signal), dynamic terms are introduced into the conventional static model.

### 2.1. Mechanistic Modeling of Quartz Flexure Accelerometers

**Figure 1:**
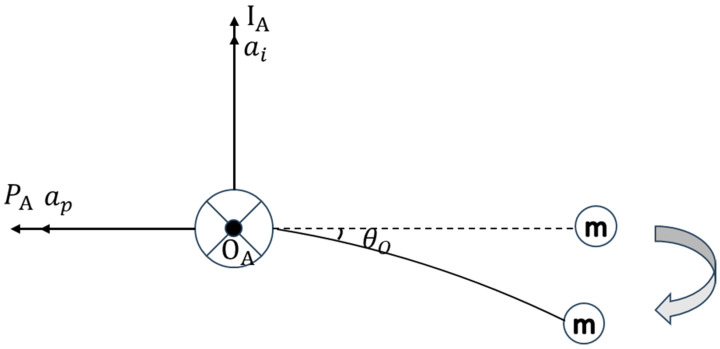
Equivalent mechanical model of the quartz flexure accelerometer.

The equivalent mechanical representation of the quartz flexure accelerometer is depicted in Figure 1. Assume that the accelerometer housing (base) undergoes accelerations $a_{i}$ and $a_{p}$ along the I-axis and P-axis, respectively. The moment of inertia of the pendulum assembly about the O-axis is denoted as $I_{o}$. The elastic stiffness of the flexure is represented by k. The damping coefficient of the fluid is μ. The signal transfer coefficient is given as $K_{S}$. The gain of the servo amplifier is denoted by $K_{A}$. The load coefficient is $K_{R}(K_{R}=\frac{1}{R_{0}+R_{T}})$, where $R_{0}$ is the sampling resistor and $R_{T}$ is the internal resistance of the torque motor. The torque motor’s scale factor is denoted as $K_{T}$. Then, according to the angular momentum theorem, L denotes the pendulum length, $M_{d}$ is the disturbance torque along the O-axis, and i represents the torque motor’s feedback current. After the external acceleration $a_{i}$ is applied, the proof mass m begins to deviate from its initial equilibrium position (represented by the dashed line), and the flexural beam starts to bend. At this stage, the mass m is subjected to the acceleration $a_{p}$, the torque generated by the torque motor, and the fluid damping force. Under the combined effect of these forces, the mass eventually moves to a new equilibrium position (represented by the solid line) at time t.

In this state, the tangent of the flexural beam at the origin forms an angle $\theta_{o}$ with respect to its original neutral position. However, since $\theta_{o}$ is relatively small (exaggerated in the figure for clarity), the curvature of the flexural beam can be neglected. The beam is therefore approximated as a straight rod of length L. The dynamic Equation is given by the following:

(2) $$I_{O}\overset{¨}{\theta_{O}}=mLa_{i}\cos(\theta_{O})-mL\sin(\theta_{O})a_{p}-\mu \dot{\theta_{O}}-k\theta_{O}-K_{T}i+M_{d}$$

Assuming a proportional (P-type) control law:

(3) $$i=K_{R}\;K_{A}\;K_{S}\;\theta_{O}$$

By neglecting the dynamic variation of the P-axis input (as $a_{p}$ changes slowly), the system can be approximated as a linear time-invariant (LTI) system. Therefore, the system response can be equivalently modeled as an initial condition problem at time zero.

### 2.2. Analysis of Dynamic Response Characteristics

Based on the second-order nonlinear differential equation of the accelerometer derived in Section 2.1, a time domain response model incorporating dynamic terms is established by analyzing the accelerometer’s response to the simple dynamic input of a ramp signal with a constant slope and intercept. This model is then simplified and reorganized to facilitate its application in practical engineering scenarios.

Let the input along the I-axis be a linear ramp signal, while the input along the P-axis is set to a constant $k_{P}$:

(4) $$\begin{array}{l} a_{i}=k_{I}\;t+b_{1}\\ a_{p}=k_{P} \end{array}$$

Given a constant P-axis input, the initial condition is as follows:

(5) $$\begin{array}{l} {\left.\;\theta_{O}\left(t\right)\right|}_{t=0}=\theta_{O}\left(0\right)\\ {\left.\frac{d\theta_{O}\left(t\right)}{dt}\right|}_{t=0}={\dot{\theta }}_{O}\left(0\right) \end{array}$$

By substituting Equations (3)–(5) into Equation (2), we obtain the following solution:

(6) $$\begin{array}{cc} \theta_{O} & =\frac{e^{-\frac{t\left(\mu -\sigma_{5}\right)}{2I_{O}}}}{\sigma_{3}}\sigma_{1}-\frac{e^{-\frac{t\left(\mu +\sigma_{5}\right)}{2I_{O}}}}{\sigma_{3}}\sigma_{2}+\frac{Lk_{I}mt}{\sigma_{4}}+\\ & \;\frac{Lm}{\sigma_{4}^{2}}(kb_{1}-\mu I+Lb_{1}k_{P}m+Kb_{1}) \end{array}$$

where $\sigma_{1}$ and $\sigma_{2}$ are parameters determined by $k_{P}$, $\theta_{O}\left(0\right)$, and ${\dot{\theta }}_{O}\left(0\right)$, and they are independent of time. ($\sigma_{1}$ and $\sigma_{2}$ are provided in Appendix A.)

$$\begin{array}{cc} K & =K_{R}\;K_{T}\;K_{A}\;K_{S}\\ \sigma_{3} & =2{\left(k+Lk_{P}m+K\right)}^{2}\cdot \\ & \sqrt{\mu^{2}-4kI_{O}-4I_{O}Lk_{P}m-4I_{O}K}\\ \sigma_{4} & =k+Lk_{P}m+K\\ \sigma_{5} & =\sqrt{\mu^{2}-4kI_{O}-4I_{O}Lk_{P}m-4I_{O}K} \end{array}$$

Based on empirical data, $\sigma_{1}$, $\sigma_{2}$, $\sigma_{3}$, and $\sigma_{4}$ are on the order of $10^{-6}$; therefore, the effects of initial conditions can be neglected under small input (≤30 g) accelerations [44,45]. Since the quartz flexure accelerometer measures the $\theta_{O}$, which is then linearly adjusted and used as the output during operation, we directly treat $\theta_{O}$ as the output E in this section. This simplification facilitates calculation and reduces the number of parameters in the model.

Consider the steady-state term in Equation (6):

(7) $$\begin{array}{cc} E & =\frac{Lk_{I}mt}{\sigma_{4}}+\frac{Lm}{\sigma_{4}^{2}}(kb_{1}-\mu I+Lk_{P}mb_{1}+Kb_{1})\\ & =-\frac{Lm\mu }{\sigma_{4}^{2}}k_{I}+\frac{Lk_{I}mt}{\sigma_{4}}+\frac{Lm(k+K+Lk_{P}m)}{\sigma_{4}^{2}}b_{1}\\ & =-\frac{Lm\mu }{\sigma_{4}^{2}}k_{I}+\frac{Lm}{\sigma_{4}}b_{1}+\frac{Lk_{I}mt}{\sigma_{4}} \end{array}$$

When $k_{p}=0$, Equation (7) can be simplified to the following:

(8) $$E=-\frac{Lm\mu }{{\left(k+K\right)}^{2}}k_{I}+\frac{Lm}{k+K}b_{1}+\frac{Lk_{I}mt}{k+K}$$

Define:

(9) $$K_{1}=\frac{Lm}{k+K}$$

(10) $$K_{2}=-\frac{Lm\mu }{{\left(k+K\right)}^{2}}=-K_{1}\frac{\mu }{k+K}$$

The steady-state term in Equation (6) can be simplified to:

(11) $$E=K_{1}k_{I}t+K_{2}k_{I}+K_{1}b_{1}=K_{1}(k_{I}t+b_{1})+\left(-K_{1}\frac{\mu }{k+K}k_{I}\right)$$

### 2.3. Development of a Calibration Model Incorporating Dynamic Terms

Based on the simplified time domain response model derived in Section 2.2, a comparison with the conventional static calibration model was conducted. By introducing dynamic terms into the existing static model to compensate for errors under dynamic inputs, a dynamic calibration model was constructed. The derived model demonstrates that, in the absence of noise, the expected steady-state error approaches zero when the system is subjected to ramp inputs containing both an intercept and a constant slope.

Under idealized assumptions—ignoring zero bias and cross-axis coupling—Equation (1) can be simplified to the following:

(12) $$a_{s}=\frac{E}{K_{1}}=a_{i}$$

For an I-axis input of $a_{i}=k_{I}t+b_{1}$, the corresponding output is as follows:

(13) $$E=K_{1}\left(k_{I}\;t+b_{1}\right)$$

Compared to Equation (12), the actual steady-state response under ideal conditions (i.e., as represented by Equation (11)) contains an error term proportional to the input derivative $k_{I}$, denoted as $K_{1}\;k_{I}$. To eliminate this error, a dynamic term is incorporated into the conventional static model, ensuring zero steady-state error when the input acceleration has a non-zero first derivative $k_{I}$ while all higher-order derivatives are zero.

The input excitation signal is considered, i.e., the derivative of Equation (4) is expressed as follows:

(14) $${\dot{a}}_{i}=k_{I}$$

Substituting the Equation (14) into Equation (11) yields the following equation:

(15) $$E=K_{1}a_{i}+\left(-K_{1}\frac{\mu }{k+K}{\dot{a}}_{i}\right)$$

In comparison with the existing static calibration model (Equation (13)), the proposed dynamic calibration model introduces a dynamic compensation term $K_{a}\;{\dot{a}}_{i}$ into the original static model, thereby enhancing accuracy under time-varying input conditions. The proposed dynamic calibration model is formulated as follows:

(16) $$E=K_{1}a_{s}=K_{1}(K_{0}+a_{i}+\varepsilon )+K_{a}\;{\dot{a}}_{i}$$

E is accelerometer output (output units);
$a_{s}$ is the indicated sensor output (m/s^2^);
$K_{1}$ is the scale factor [output units/(m/s^2^)];
ε is random noise (m/s^2^);
$K_{0}$ is the bias (m/s^2^);
$K_{a}$ is the dynamic compensation coefficient (s);
$a_{i}$ is the acceleration along sensor IA (m/s^2^);
${\dot{a}}_{i}$ is the time derivative of acceleration along sensor IA (m/s^3^).

Theoretically, the dynamic compensation coefficient $K_{a}$ can be expressed as follows:

(17) $$K_{a}=-\frac{\mu K_{1}}{k+K}$$

## 3. Performance Analysis of the Dynamic Model

Using theoretical outputs derived from actual instrument parameters, we compare the error characteristics between the dynamic and static models. Subsequently, the relative dynamic error index $\varepsilon_{d}$ is used to quantify the accuracy improvement provided by the dynamic model over the static model under dynamic input conditions. The results indicate that, under sinusoidal excitation, the accelerometer generates a sinusoidal response with a phase lag and minor amplitude variation within the typical operating frequency range. Compared with the static model, the dynamic model exhibits superior amplitude and phase compensation, thereby achieving higher overall accuracy.

### 3.1. Analysis of Accelerometer Model Output

Applying the Laplace transform to Equation (2), the expression can be rearranged as follows:

(18) $$\begin{array}{c} I_{O}s^{2}L\{\theta_{O}(t)\}-I_{O}\left(s\theta_{O}(0)+{\left.\frac{d\theta_{O}(t)}{dt}\right|}_{t=0}\right)\\ =(-k-\mu s)L\{\theta_{O}(t)\}+\mu \theta_{O}(0)-K_{T}L\{i(t)\}+\\ L\{M_{d}(t)\}+LmL\{\cos(\theta_{O}(t))a_{i}(t)\}-LmL\{\sin(\theta_{O}(t))a_{p}(t)\} \end{array}$$

Substituting the designed control law D(s), i.e., Equation (3), the following is obtained:

(19) $$\begin{array}{c} I_{O}s^{2}L\{\theta_{O}(t)\}-I_{O}\left(s\theta_{O}(0)+{\left.\frac{d\theta_{O}(t)}{dt}\right|}_{t=0}\right)\\ =(-k-\mu s-K)L\{\theta_{O}(t)\}+\mu \theta_{O}(0)+\\ L\{M_{d}(t)\}+LmL\{\cos(\theta_{O}(t))a_{i}(t)\}-LmL\{\sin(\theta_{O}(t))a_{p}(t)\} \end{array}$$

When the initial conditions are assumed to be zero and external disturbances are neglected, the governing equation simplifies to the following equation:

(20) $$\left(I_{O}s^{2}+\mu s+k+La_{p,\mathrm{val}}m+K)L\{\theta_{O}(t)\}=LmL\{a_{i}(t)\right\}$$

**Figure 2:**
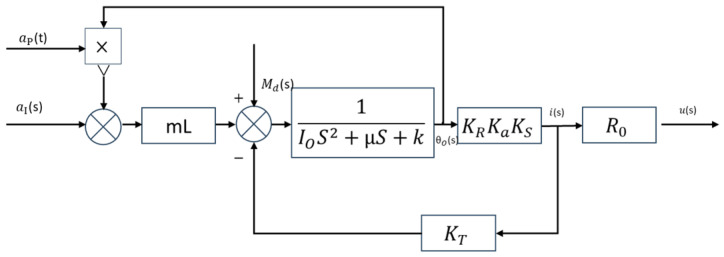
Closed-loop block diagram of the accelerometer.

Based on Equation (19), the block diagram of the accelerometer is derived, as illustrated in Figure 2. The transfer function is then derived as follows:

(21) $$\frac{L\{\theta_{O}(t)\}}{L\{a_{i}(t)\}}=\frac{Lm}{I_{O}s^{2}+\mu s+k+La_{p,\mathrm{val}}m+K}$$

When transforming to the output voltage circuit U:

(22) $$\frac{U(s)}{a_{i}(s)}=\frac{LmK_{R}K_{A}K_{S}}{I_{O}s^{2}+\mu s+k+La_{p,\mathrm{val}}m+K}$$

By neglecting the P-axis input, the system simplifies to a second-order dynamic model, whose amplitude frequency response is characterized by the following:

(23) $$|H(j\omega )|=\frac{LmK_{R}K_{A}K_{s}}{\sqrt{{\left(k+K-I_{O}\omega^{2}\right)}^{2}+{\left(\mu \omega \right)}^{2}}}$$

The phase frequency response is as follows:

(24) $$∠H(j\omega )=-\arctan\left(\frac{\mu \omega }{k+K-I_{O}\omega^{2}}\right)$$

### 3.2. Dynamic Model Output Analysis

By substituting actual accelerometer parameters, we assess the accuracy of the dynamic error model under dynamic input conditions.

The input signal is set as the following sinusoidal function:

(25) $$a_{i}=\sin(\omega \;t+b)$$

Under this condition, the dynamic model yields the following output:

(26) $$\left(\sqrt{\frac{\mu^{2}\omega^{2}}{{\left(k+K\right)}^{2}}+1}\right)\cdot \sin\left(b+\arctan\left(\frac{\mu \omega }{k+K}\right)+t\omega \right)$$

Using measured parameters from a specific accelerometer, we compare the calibration performance of the static and dynamic models under representative input conditions [44,45]:

$$\begin{array}{l} \mu =1.69\times 10^{-4}\;N\cdot m\cdot s/\mathrm{rad}\\ k=6.28\times 10^{-4}\;N\cdot m/\mathrm{rad}\\ K=0.2547\\ I_{O}=7.01\times 10^{-8}\;kg\cdot m^{2}\\ L=6.5\times 10^{-3}\;m\\ m=1.659\times 10^{-3}\;kg \end{array}$$

**Figure 3:**
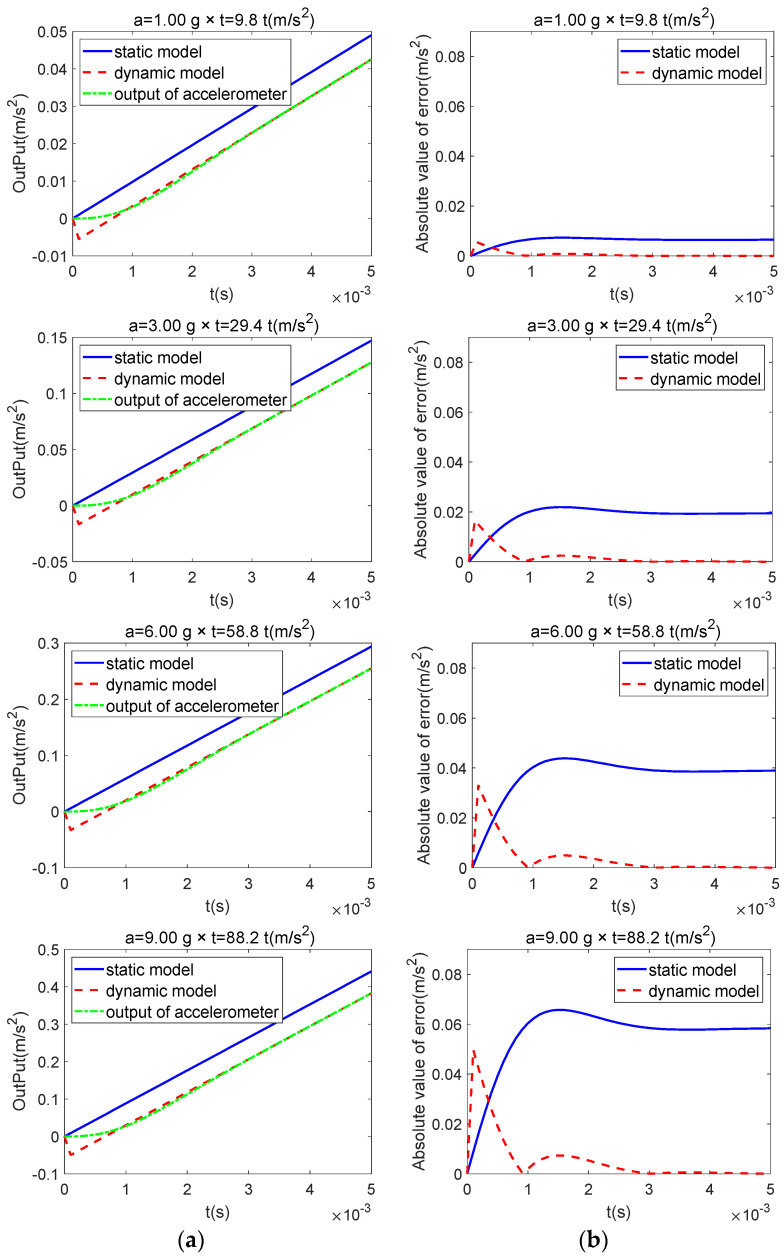
Comparison of the static model, dynamic model, and actual output under ramp input: (**a**) output; (**b**) error.

For ramp inputs with varying slopes along the I-axis, Figure 3a illustrates the actual accelerometer output alongside the ideal outputs of both the dynamic and static models, while Figure 3b presents the corresponding absolute errors relative to the input (only the initial portion after the ramp input is shown, as subsequent outputs are straight lines). The results show that, under a ramp input, the accelerometer produces a steady-state linear output with a slope matching that of the input after a brief transient response. The static model likewise yields a straight line with the same slope as the actual accelerometer output; however, it shows a constant offset relative to the accelerometer output, and this offset increases with the input slope. In contrast, the proposed dynamic model yields an output identical to the accelerometer’s steady-state response, namely a straight line with the same slope and intercept as the accelerometer output. This result is consistent with the derivations in Section 2, which predict that the static model has a steady-state error under ramp inputs, while the dynamic model eliminates this error by introducing a dynamic compensation term $K_{a}\;{\dot{a}}_{i}$, thereby achieving zero steady-state error for ramp inputs.

**Figure 4:**
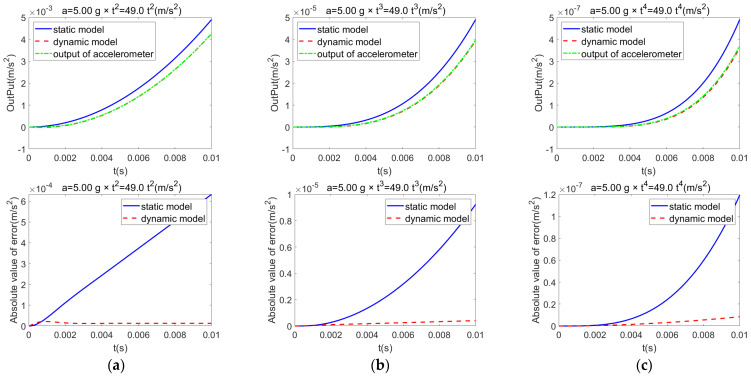
Comparison of the static model, dynamic model, and actual output under low-order polynomial inputs: (**a**) quadratic; (**b**) cubic; (**c**) quartic.

When the I-axis input is a low-order polynomial and the P-axis input is set to zero, the dynamic model exhibits excellent tracking performance with respect to the actual accelerometer output, and it significantly outperforms the static model. Figure 4 presents the actual accelerometer output, as well as the outputs and corresponding errors of the static and dynamic models under the following low-order polynomial inputs: (a) quadratic, (b) cubic, and (c) quartic inputs. As the polynomial order increases, the tracking performance of the static model gradually degrades, whereas the dynamic model consistently maintains a low error magnitude.

**Figure 5:**
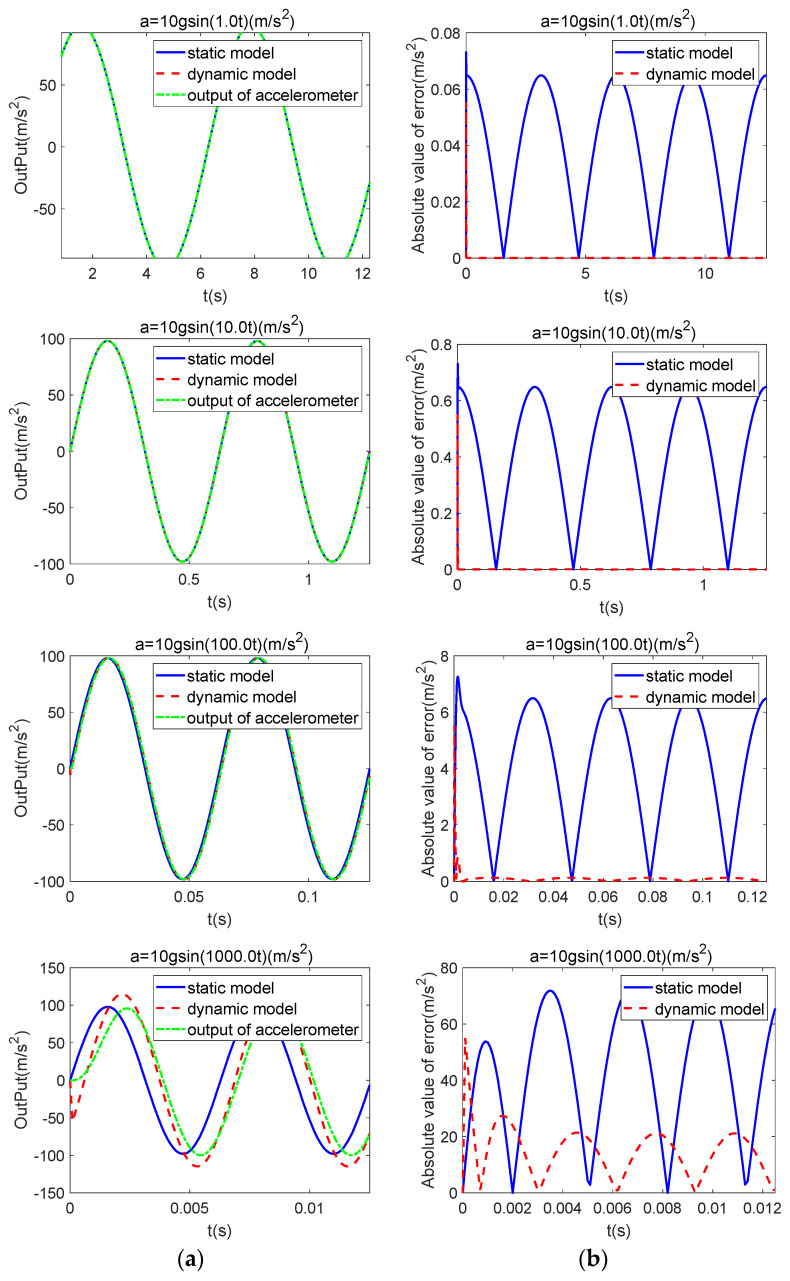
Comparison of the static model, dynamic model, and actual output under sinusoidal input: (**a**) output; (**b**) error.

With a sinusoidal input along the I-axis, having an amplitude of 10 g and a frequency range of 1~1000 rad/s, and with the P-axis input set to zero, Figure 5a displays the actual accelerometer output alongside those of the ideal dynamic and static models; the corresponding absolute input-output deviations are illustrated in Figure 5b.

It is evident that when a sinusoidal signal is applied, the accelerometer generates a sinusoidal output with a phase lag and minor amplitude variation over the standard operating frequency range. Compared with the static model, the dynamic model offers moderate amplitude compensation and substantially better phase correction. This leads to a clear improvement in overall accuracy.

**Figure 6:**
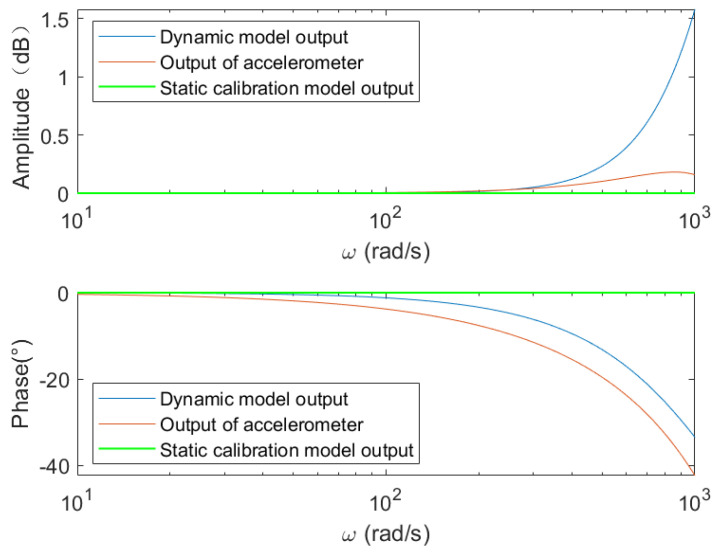
Amplitude and phase frequency response characteristics.

Figure 6 presents the amplitude and phase frequency characteristics of the accelerometer and both dynamic and static models. Compared to the static model (which has zero gain and phase delay), the amplitude response aligns well at low frequencies, while the phase response shows substantial improvement. As frequency increases, the amplitude response worsens, but the improvement in phase remains evident.

However, according to the calculation based on Equation (26) and the results shown in Figure 6, the output amplitude of the proposed dynamic model under a sinusoidal input is $\left(\sqrt{\frac{\mu^{2}w^{2}}{{\left(k+K\right)}^{2}}+1}\right)$. It is evident that $\left(\sqrt{\frac{\mu^{2}w^{2}}{{\left(k+K\right)}^{2}}+1}\right)\geq 1$, and therefore, the amplitude of the input signal will be amplified. When ω is relatively small, $\left(\sqrt{\frac{\mu^{2}w^{2}}{{\left(k+K\right)}^{2}}+1}\right)$, approaches 1, indicating good amplitude tracking performance. Since the phase tracking performance is significantly better than that of the static model, the overall accuracy is notably improved. However, as the frequency increases, the output amplification factor correspondingly increases. In this case, although the phase tracking performance remains far superior to that of the static model, the amplitude tracking performance deteriorates. At sufficiently high input frequencies, the performance may even become worse than that of the static model. Therefore, the proposed model is not suitable for high-frequency testing, particularly when the angular frequency of the input signal satisfies ω≥1000(rad/s).

### 3.3. Model Performance Assessment

Traditional time domain performance indicators for accelerometer dynamic characteristics—such as overshoot, settling time, oscillation count, rise time, and peak time—are single-metric measures that can only reflect isolated aspects of the system’s deterministic behavior. To comprehensively evaluate the accelerometer’s dynamic characteristics, a relative dynamic integral error metric $\varepsilon_{d}$ is defined [1]. It is specifically defined as follows:

(27) $$\varepsilon \left(t\right)=\frac{x(t)-y(t)}{A}$$

(28) $$\varepsilon_{d}=\sqrt{\frac{\int_{0}^{T}\varepsilon {\left(t\right)}^{2}dt}{T}}$$

where *T* is the test duration, ε(t) is the relative dynamic error, $x\left(t\right)$ and $y\left(t\right)$ are the input and system output signals, respectively, and A is the amplitude of $x\left(t\right)$.

The relative dynamic error formulation is used to eliminate the influence of input amplitude on the magnitude of the metric.

**Figure 7:**
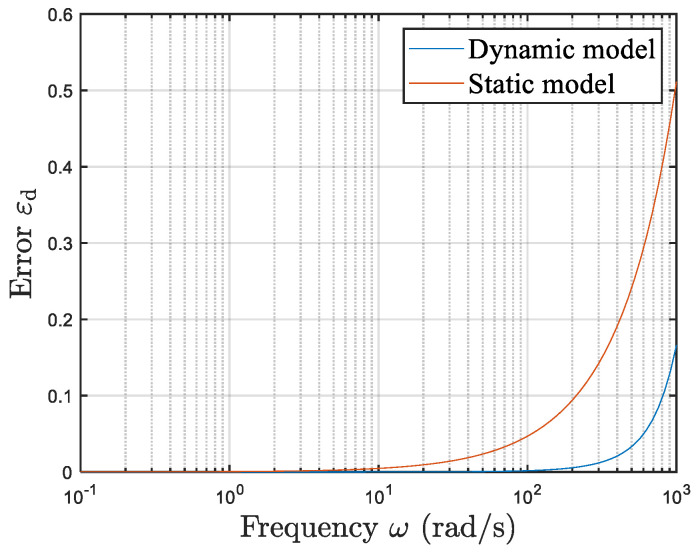
Frequency-dependent dynamic error comparison between static and dynamic models.

Table 1 presents the error values at different frequencies (with T set to 10 times the signal period during the simulation tests). Figure 7 shows the error curves of the static and dynamic models at different frequencies. The results clearly indicate that, within typical operating frequencies, the dynamic model markedly outperforms the static model in terms of accuracy, often by several orders of magnitude.

**Figure 8:**
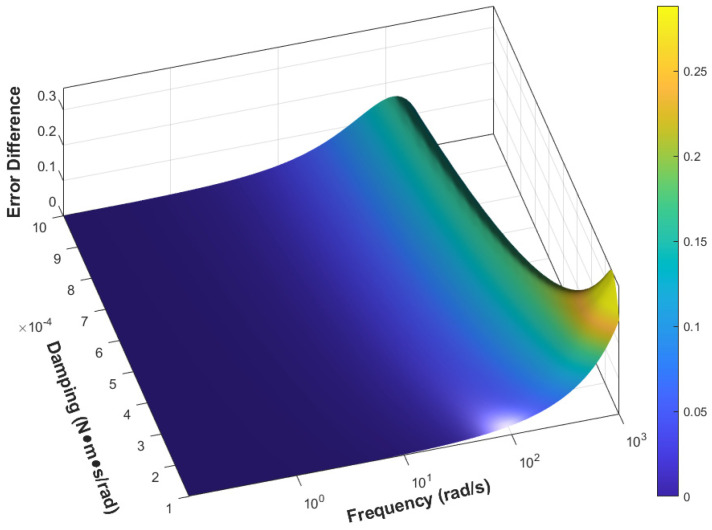
Error improvement ratio damping–frequency.

Considering that different types of accelerometers have varying damping coefficients, Figure 8 illustrates the improvement in the dynamic integral error metric $\varepsilon_{d}$ of the dynamic model relative to the static model across typical damping values and input frequencies. It can be observed that, in most cases, the dynamic model yields lower error than the static model for typical quartz flexure accelerometers operating within standard frequency ranges, and the degree of error reduction increases with frequency. After reaching a peak, the improvement diminishes due to the degradation of the amplitude–frequency enhancement effect. The peak point varies with different damping values; as damping increases, the frequency corresponding to the peak decreases. In the low-damping and low-frequency region, although the dynamic integral error metric improves by orders of magnitude, its actual impact remains negligible due to the initially small error values.

## 4. Design of Calibration Method

By analyzing the coordinate transformations of the test system and external excitation sources, the actual specific force along each axis of the accelerometer is derived, enabling the elimination of the effects of gravitational acceleration, Coriolis acceleration, and other external disturbances during the calibration process. The error sources are thoroughly analyzed to support the evaluation of expected calibration performance and to provide a theoretical foundation for reducing dominant error components through mathematical or engineering approaches, thereby improving calibration accuracy.

Based on the proposed error model and the aforementioned analysis, a simplified calibration method is developed using a dual-axis precision centrifuge. This method applies sinusoidal inputs with varying frequencies to the accelerometer and adopts least squares estimation to identify the parameters of the calibration model. Additionally, full-cycle integration is employed to suppress periodic errors. A complete calibration procedure is thus established for the simultaneous identification of both scale factors and dynamic coefficients of the accelerometer.

### 4.1. Error Source Analysis and Coordinate Transformation

The coordinate transformation and error sources of the test system are analyzed and calculated to obtain the transformation matrix, which is then used to compute the accurate specific force input to the accelerometer and to analyze calibration errors in subsequent sections.

**Figure 9:**
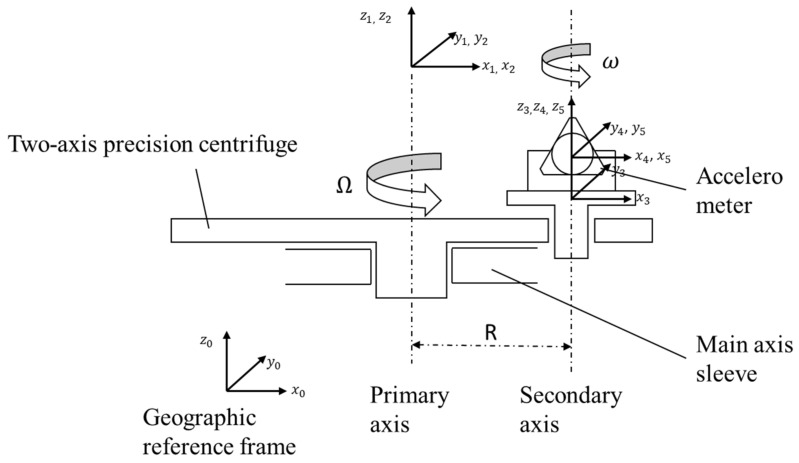
Dual-axis precision centrifuge schematic diagram.

Figure 9 shows the structure of the dual-axis precision centrifuge.

The static error sources of the dual-axis precision centrifuge include two-dimensional perpendicularity errors of the main axis $\left(\Delta \theta_{x0},\Delta \theta_{y0}\right)$ and the static radius measurement error ΔR; the parallelism deviation between the azimuth axis and the main shaft $\left(\Delta \theta_{x2},\Delta \theta_{y2}\right)$, the installation base misalignment of the accelerometer $\left(\Delta \theta_{x3},\Delta \theta_{y3},\Delta \theta_{z3}\right)$, and the eccentricity errors (Δx, Δy, Δz); as well as the misalignment between the accelerometer casing and its internal pendulum structure $\left(\Delta \theta_{x4},\Delta \theta_{y4},\Delta \theta_{z4}\right)$. The dynamic error sources include rotational inaccuracies caused by shaft motion, specifically radial runout errors $\left(\Delta x_{1}\left(\alpha \right),\Delta y_{1}\left(\alpha \right)\right)$, axial wobble $\left(\Delta z_{1}\left(\alpha \right)\right)$, tilt angle errors $\left(\Delta \theta_{x1}\left(\alpha \right),\Delta \theta_{y1}\left(\alpha \right)\right)$, and the centrifuge of dynamic radius $R_{d}$. Table 2 presents the coordinate system transformations, incorporating all the aforementioned error sources of the dual-axis precision centrifuge. During the time interval $\left(0,\;t\right)$, the primary shaft rotates through an angle *α*, where the local latitude is denoted by L.

where:

rot(coordinate axis,angle) and $\mathrm{tran}(t_{x},t_{y},t_{z})$ are defined as the rotation matrices and translation matrices, rot(coordinate axis,angle) respectively:

$$\begin{array}{l} \mathrm{rot}(x,\alpha )=\left[\begin{array}{cccc} 1 & 0 & 0 & 0\\ 0 & \cos\alpha & -\sin\alpha & 0\\ 0 & \sin\alpha & \cos\alpha & 0\\ 0 & 0 & 0 & 1 \end{array}\right]\;\mathrm{rot}(y,\beta )=\left[\begin{array}{cccc} \cos\beta & 0 & \sin\beta & 0\\ 0 & 1 & 0 & 0\\ -\sin\beta & 0 & \cos\beta & 0\\ 0 & 0 & 0 & 1 \end{array}\right]\;\\ \mathrm{rot}(z,\gamma )=\left[\begin{array}{cccc} \cos\gamma & -\sin\gamma & 0 & 0\\ \sin\gamma & \cos\gamma & 0 & 0\\ 0 & 0 & 1 & 0\\ 0 & 0 & 0 & 1 \end{array}\right] \end{array}$$

When the angles are small, they can be approximated as follows:

$$\mathrm{rot}(x,\alpha )=\left[\begin{array}{cccc} 1 & 0 & 0 & 0\\ 0 & 1 & -\alpha & 0\\ 0 & \alpha & 1 & 0\\ 0 & 0 & 0 & 1 \end{array}\right]\;\mathrm{rot}(y,\beta )=\left[\begin{array}{cccc} 1 & 0 & \beta & 0\\ 0 & 1 & 0 & 0\\ -\beta & 0 & 1 & 0\\ 0 & 0 & 0 & 1 \end{array}\right]\;\mathrm{rot}(z,\gamma )=\left[\begin{array}{cccc} 1 & -\gamma & 0 & 0\\ \gamma & 1 & 0 & 0\\ 0 & 0 & 1 & 0\\ 0 & 0 & 0 & 1 \end{array}\right]$$

$\mathrm{tran}(t_{x},t_{y},t_{z})$ is defined as follows:

$$\mathrm{tran}(t_{x},t_{y},t_{z})=\left[\begin{array}{cccc} 1 & 0 & 0 & t_{x}\\ 0 & 1 & 0 & t_{y}\\ 0 & 0 & 1 & t_{z}\\ 0 & 0 & 0 & 1 \end{array}\right]$$

Accordingly, it can be derived that:

$$\begin{array}{cc} T_{1}^{0} & =\mathrm{rot}(x_{0},\Delta \theta_{x0})\cdot \mathrm{rot}(y_{0},\Delta \theta_{y0})\\ & =\left[\begin{array}{cccc} 1 & 0 & \Delta \theta_{y0} & 0\\ 0 & 1 & -\Delta \theta_{x0} & 0\\ -\Delta \theta_{y0} & \Delta \theta_{x0} & 1 & 0\\ 0 & 0 & 0 & 1 \end{array}\right]\\ T_{2}^{1} & =\mathrm{tran}\left(\Delta x_{1}(\alpha ),\Delta y_{1}(\alpha ),\Delta z_{1}(\alpha )\right)\cdot \mathrm{rot}(x_{1},\Delta \theta_{x1}(\alpha ))\cdot \mathrm{rot}(y_{1},\Delta \theta_{y1}(\alpha ))\cdot \mathrm{rot}(z_{1},\alpha )\\ & =\left[\begin{array}{cccc} 1 & 0 & 0 & \Delta x_{1}(\alpha )\\ 0 & 1 & 0 & \Delta y_{1}(\alpha )\\ 0 & 0 & 1 & \Delta z_{1}(\alpha )\\ 0 & 0 & 0 & 1 \end{array}\right]\cdot \left[\begin{array}{cccc} 1 & 0 & \Delta \theta_{y1}(\alpha ) & 0\\ 0 & 1 & -\Delta \theta_{x1}(\alpha ) & 0\\ -\Delta \theta_{y1}(\alpha ) & \Delta \theta_{x1}(\alpha ) & 1 & 0\\ 0 & 0 & 0 & 1 \end{array}\right]\cdot \left[\begin{array}{cccc} \cos\alpha & -\sin\alpha & 0 & 0\\ \sin\alpha & \cos\alpha & 0 & 0\\ 0 & 0 & 1 & 0\\ 0 & 0 & 0 & 1 \end{array}\right]\\ T_{3}^{2} & =\mathrm{tran}(R+\Delta R,0,h)\cdot \mathrm{rot}(x_{2},\Delta \theta_{x2})\cdot \mathrm{rot}(y_{2},\Delta \theta_{y2})\cdot \mathrm{rot}(z_{2},\beta )\\ & =\left[\begin{array}{cccc} 1 & 0 & 0 & R+\Delta R\\ 0 & 1 & 0 & 0\\ 0 & 0 & 1 & h\\ 0 & 0 & 0 & 1 \end{array}\right]\cdot \left[\begin{array}{cccc} 1 & 0 & \Delta \theta_{y2} & 0\\ 0 & 1 & -\Delta \theta_{x2} & 0\\ -\Delta \theta_{y2} & \Delta \theta_{x2} & 1 & 0\\ 0 & 0 & 0 & 1 \end{array}\right]\cdot \left[\begin{array}{cccc} \cos\beta & -\sin\beta & 0 & 0\\ \sin\beta & \cos\beta & 0 & 0\\ 0 & 0 & 1 & 0\\ 0 & 0 & 0 & 1 \end{array}\right]\\ T_{4}^{3} & =\mathrm{rot}(x_{3},\Delta \theta_{x3})\cdot \mathrm{rot}(y_{3},\Delta \theta_{y3})\cdot \mathrm{rot}(z_{3},\Delta \theta_{z3})\cdot \mathrm{tran}(\Delta x,\Delta y,\Delta z)\\ & =\left[\begin{array}{cccc} 1 & -\Delta \theta_{z3} & \Delta \theta_{y3} & \Delta x\\ \Delta \theta_{z3} & 1 & -\Delta \theta_{x3} & \Delta y\\ -\Delta \theta_{y3} & \Delta \theta_{x3} & 1 & \Delta z\\ 0 & 0 & 0 & 1 \end{array}\right]\\ T_{5}^{4} & =\mathrm{rot}(x_{4},\Delta \theta_{x4})\cdot \mathrm{rot}(y_{4},\Delta \theta_{y4})\cdot \mathrm{rot}(z_{4},\Delta \theta_{z4})\\ & =\left[\begin{array}{cccc} 1 & -\Delta \theta_{z4} & \Delta \theta_{y4} & 0\\ \Delta \theta_{z4} & 1 & -\Delta \theta_{x4} & 0\\ -\Delta \theta_{y4} & \Delta \theta_{x4} & 1 & 0\\ 0 & 0 & 0 & 1 \end{array}\right] \end{array}$$

The final homogeneous transformation matrix is given by the following:

(29) $$T_{1}^{0}\;T_{2}^{1}\;T_{3}^{2}\;T_{4}^{3}\;T_{5}^{4}=\left(\begin{array}{cc} \alpha & \beta \\ 0 & 1 \end{array}\right)$$

where:

$$\begin{array}{l} \alpha =A_{1}\;A_{2}\;A_{3}\;A_{4}\;A_{5}\;A_{6}\;A_{7}\\ \beta =A_{1}\;A_{2}\;A_{3}\;A_{4}\;A_{5}\;A_{6}\;D_{3}+A_{1}\;A_{2}\;A_{3}\;D_{2}+A_{1}\;D_{1} \end{array}$$

### 4.2. Specific Force Input

Based on the previously derived coordinate transformation matrix, the precise input from each excitation source along each axis of the accelerometer is calculated, resulting in the composite acceleration input for each axis. The primary excitation sources considered include gravitational acceleration, the centripetal and tangential accelerations generated by the centrifuge, and Coriolis acceleration.

#### 4.2.1. Gravitational Acceleration Components on Each Axis

The gravitational acceleration vector is expressed as ${\left[\begin{array}{ccc} 0 & 0 & g \end{array}\right]}^{T}$ in the geographic coordinate system, and is transformed into the accelerometer coordinate system as follows:

(30) $${\left(\begin{array}{ccc} a_{\mathrm{ig}} & a_{\mathrm{pg}} & a_{\mathrm{og}} \end{array}\right)}^{T}={\left(A_{1}\;A_{2}\;A_{3}\;A_{4}\;A_{5}\;A_{6}\;A_{7}\right)}^{T}{\left[\begin{array}{ccc} 0 & 0 & g \end{array}\right]}^{T}$$

By neglecting second-order and higher-order small terms, the equation simplifies to the following:

(31) $$\begin{array}{cc} a_{\mathrm{ig}} & =-[\Delta \theta_{y3}+\Delta \theta_{y4}+\Delta \theta_{y0}\cos(\alpha +\beta )+\Delta \theta_{y1}(\alpha )\cos(\alpha +\beta )-\Delta \theta_{x0}\sin(\alpha +\beta )-\\ & \;\Delta \theta_{x1}(\alpha )\sin(\alpha +\beta )+\Delta \theta_{y2}\cos\beta -\Delta \theta_{x2}\sin\beta ]g\\ a_{\mathrm{pg}} & =g(\Delta \theta_{x3}+\Delta \theta_{x4}+\Delta \theta_{x0}\cos(\alpha +\beta )+\Delta \theta_{x1}(\alpha )\cos(\alpha +\beta )+\Delta \theta_{y0}\sin(\alpha +\beta )+\\ & \;\Delta \theta_{y1}(\alpha )\sin(\alpha +\beta )+\Delta \theta_{x2}\cos\beta +\Delta \theta_{y2}\sin\beta )\\ a_{\mathrm{og}} & =\;g \end{array}$$

#### 4.2.2. Components of Centripetal and Tangential Acceleration in the Accelerometer Coordinate Axes

During the time interval $\left(0,t\right)$, the primary and secondary shafts rotate by angles α and β, respectively.

Using the pose transformation matrix and projecting onto the accelerometer’s pendulum coordinate system, the accelerations applied by the precision centrifuge to the input, output, and pendulum axes can be determined as the following:

(32) $$D=A_{1}\;A_{2}\;A_{3}\;A_{4}\;A_{5}\;A_{6}\;A_{7}\;D_{3}+A_{1}\;A_{2}\;A_{3}\;D_{2}+A_{1}\;D_{1}$$

Taking the second derivative of it with respect to time yields:

(33) $$\frac{d^{2}D}{dt^{2}}=A_{1}\;A_{2}\;{\ddot{A}}_{3}(A_{4}\;A_{5}\;A_{6}\;A_{7}\;D_{3}+D_{2})$$

By using the pose transformation matrix and projecting back to the accelerometer pendulum coordinate system, the accelerations applied by the precision centrifuge to the input, output, and pendulum axes of the accelerometer can be obtained as follows:

(34) $$\begin{array}{cc} a_{i\omega } & =\Delta R\;\ddot{\alpha }\;\sin\beta -\Delta x\;{\dot{\alpha }}^{2}-\Delta R\;{\dot{\alpha }}^{2}\;\cos\beta -R\;{\dot{\alpha }}^{2}\cos\beta -\Delta y\;\ddot{\alpha }+R\;\ddot{\alpha }\;\sin\beta +\\ & \;\Delta \theta_{z3}\;R\;\ddot{\alpha }\cos\beta +\Delta \theta_{z4}\;R\;\ddot{\alpha }\;\cos\beta +\Delta \theta_{z3}\;R\;{\dot{\alpha }}^{2}\;\sin\beta +\Delta \theta_{z4}\;R\;{\dot{\alpha }}^{2}\;\sin\beta \\ a_{p\omega } & =\Delta x\;\ddot{\alpha }-\Delta y\;{\dot{\alpha }}^{2}+\Delta R\;{\dot{\alpha }}^{2}\;\sin\beta +R\;{\dot{\alpha }}^{2}\;\sin\beta +\Delta R\;\ddot{\alpha }\;\cos\beta +R\;\ddot{\alpha }\;\cos\beta -\\ & \;\Delta \theta_{z3}\;R\;\ddot{\alpha }\;\sin\beta -\Delta \theta_{z4}\;R\;\ddot{\alpha }\;\sin\beta +\Delta \theta_{z3}\;R\;{\dot{\alpha }}^{2}\;\cos\beta +\Delta \theta_{z4}\;R\;{\dot{\alpha }}^{2}\;\cos\beta \\ a_{o\omega } & =-R(\Delta \theta_{x2}\;\ddot{\alpha }+\Delta \theta_{y2}\;{\dot{\alpha }}^{2}+\Delta \theta_{y3}\;{\dot{\alpha }}^{2}\;\cos\beta +\Delta \theta_{y4}\;{\dot{\alpha }}^{2}\;\cos\beta +\Delta \theta_{x3}\;{\dot{\alpha }}^{2}\;\sin\beta +\\ & \;\Delta \theta_{x4}\;{\dot{\alpha }}^{2}\;\sin\beta +\Delta \theta_{x3}\;\ddot{\alpha }\;\cos\beta +\Delta \theta_{x4}\;\ddot{\alpha }\;\cos\beta -\Delta \theta_{y3}\;\ddot{\alpha }\;\sin\beta -\Delta \theta_{y4}\;\ddot{\alpha }\;\sin\beta ) \end{array}$$

#### 4.2.3. Coriolis Acceleration Components Along the Three Axes of the Accelerometer Coordinate System

Let the local latitude be L and the Earth’s angular velocity be $\omega_{ie}$. Since the Coriolis acceleration is small, only the nominal value is considered, and small error terms are ignored.

(35) $$\left(\begin{array}{c} a_{\mathrm{ic}}\\ a_{\mathrm{pc}}\\ a_{\mathrm{oc}} \end{array}\right)=2{\left(A_{3}\;A_{5}\right)}^{T}\left\{\left(\begin{array}{c} 0\\ \omega_{\mathrm{ie}}\;\cos L\\ \omega_{\mathrm{ie}}\;\sin L \end{array}\right)\times \left(\begin{array}{c} -R\;\dot{\alpha }\;\sin\alpha \\ R\;\dot{\alpha }\;\cos\alpha \\ 0 \end{array}\right)\right\}$$

The resulting expressions are:

(36) $$\begin{array}{c} a_{\mathrm{ic}}=-2\;R\;\dot{\alpha }\;\omega_{\mathrm{ie}}\;(\sin L\;\cos\beta )\\ a_{\mathrm{pc}}=2\;R\;\dot{\alpha }\;\omega_{\mathrm{ie}}(\sin L\;\sin\beta )\\ a_{\mathrm{oc}}=-2\;R\;\dot{\alpha }\;\omega_{\mathrm{ie}}(-\cos L\;\sin\alpha ) \end{array}$$

#### 4.2.4. Composite Specific Force

**Figure 10:**
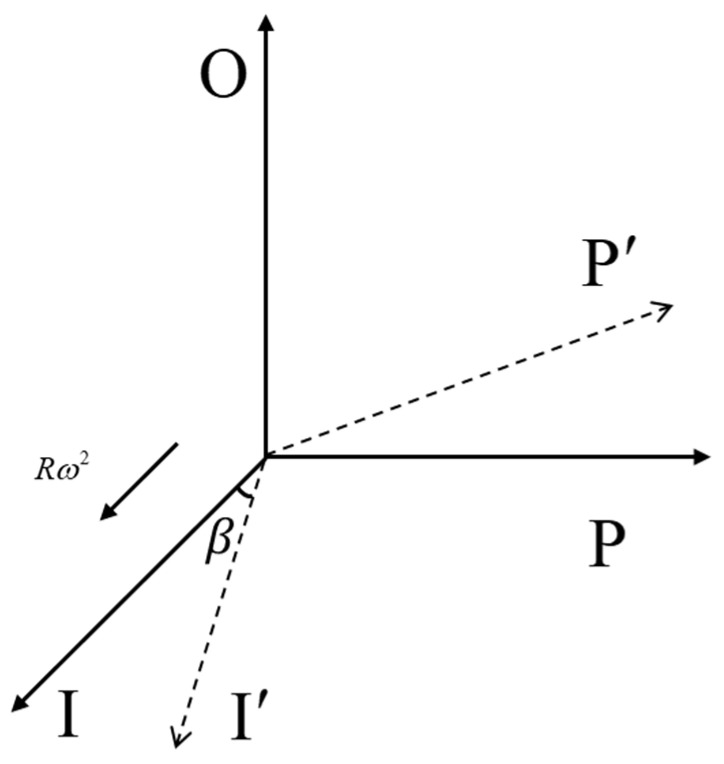
Installation configuration of the accelerometer on the dual-axis precision centrifuge.

In the initial installation shown in Figure 10, the I-axis coincides with $X_{5}$ toward the primary shaft, OA with $Z_{5}$, and PA with $Y_{5}$.

Consequently, the final input accelerations along the three accelerometer axes are given as follows:

(37) $$\begin{array}{cc} a_{i} & =\Delta R\;\ddot{\alpha }\;\sin\beta -\Delta \theta_{y3}\;g-\Delta \theta_{y4}\;g-\Delta x\;{\dot{\alpha }}^{2}-\Delta R\;{\dot{\alpha }}^{2}\;\cos\beta -R\;{\dot{\alpha }}^{2}\;\cos\beta -\\ & \;\Delta \theta_{y2}\;g\;\cos\beta -\Delta y\;\ddot{\alpha }+\Delta \theta_{x2}\;g\;\sin\beta +R\;\ddot{\alpha }\;\sin\beta +\Delta \theta_{z3}\;R\;\ddot{\alpha }\;\cos\beta +\\ & \;\Delta \theta_{z4}\;R\;\ddot{\alpha }\;\cos\beta +\Delta \theta_{z3}\;R\;{\dot{\alpha }}^{2}\sin\beta +\Delta \theta_{z4}\;R\;{\dot{\alpha }}^{2}\;\sin\beta -\Delta \theta_{y0}\;g\;\cos\alpha \;\cos\beta \\ & \;-\Delta \theta_{y1}(\alpha )\;g\;\cos\alpha \;\cos\beta +\Delta \theta_{x0}\;g\;\cos\alpha \;\sin\beta +\Delta \theta_{x0}\;g\;\cos\beta \;\sin\alpha +\\ & \;\Delta \theta_{x1}(\alpha )\;g\;\cos\alpha \;\sin\beta +\Delta \theta_{x1}(\alpha )\;g\;\cos\beta \;\sin\alpha +\Delta \theta_{y0}\;g\;\sin\alpha \;\sin\beta +\\ & \;\Delta \theta_{y1}(\alpha )\;g\;\sin\alpha \;\sin\beta -2\;R\;\dot{\alpha }\;\omega_{\mathrm{ie}}\;\sin L\;\cos\beta -2\;\Delta \theta_{y4}\;R\;\dot{\alpha }\;\omega_{\mathrm{ie}}\;\cos L\;\sin\alpha +\\ & \;2\;\Delta \theta_{z4}\;R\;\dot{\alpha }\;\omega_{\mathrm{ie}}\;\sin L\;\sin\beta \end{array}$$

(38) $$\begin{array}{cc} a_{p} & =\Delta x\;\ddot{\alpha }+\Delta \theta_{x3}\;g+\Delta \theta_{x4}\;g-\Delta y\;{\dot{\alpha }}^{2}+\Delta R\;{\dot{\alpha }}^{2}\;\sin\beta +R\;{\dot{\alpha }}^{2}\;\sin\beta +\Delta R\;\ddot{\alpha }\;\cos\beta +\\ & \;\Delta \theta_{x2}\;g\;\cos\beta +R\;\ddot{\alpha }\;\cos\beta +\Delta \theta_{y2}\;g\;\sin\beta -\Delta \theta_{z3}\;R\;\ddot{\alpha }\;\sin\beta -\Delta \theta_{z4}\;R\;\ddot{\alpha }\;\sin\beta +\\ & \;\Delta \theta_{z3}\;R\;{\dot{\alpha }}^{2}\;\cos\beta +\Delta \theta_{z4}\;R\;{\dot{\alpha }}^{2}\;\cos\beta +\Delta \theta_{x0}\;g\;\cos\alpha \;\cos\beta +\Delta \theta_{x1}(\alpha )\;g\;\cos\alpha \;\cos\beta +\\ & \;\Delta \theta_{y0}\;g\;\cos\alpha \;\sin\beta +\Delta \theta_{y0}\;g\;\cos\beta \;\sin\alpha +\Delta \theta_{y1}(\alpha )\;g\;\cos\alpha \;\sin\beta +\\ & \;\Delta \theta_{y1}(\alpha )\;g\;\cos\beta \;\sin\alpha -\Delta \theta_{x0}\;g\;\sin\alpha \;\sin\beta -\Delta \theta_{x1}(\alpha )\;g\;\sin\alpha \;\sin\beta +\\ & \;2R\;\dot{\alpha }\;\omega_{\mathrm{ie}}\;\sin L\;\sin\beta +2\Delta \theta_{x4}\;R\;\dot{\alpha }\;\omega_{\mathrm{ie}}\;\cos L\;\sin\alpha +2\Delta \theta_{z4}\;R\;\dot{\alpha }\;\omega_{\mathrm{ie}}\;\sin L\;\cos\beta \end{array}$$

(39) $$\begin{array}{cc} a_{o} & =g-\Delta \theta_{x2}\;R\;\ddot{\alpha }-\Delta \theta_{y2}\;R\;{\dot{\alpha }}^{2}-\Delta \theta_{x3}\;R\;\ddot{\alpha }\;\cos\beta -\Delta \theta_{x4}\;R\;\ddot{\alpha }\;\cos\beta +\\ & \;\Delta \theta_{y3}\;R\;\ddot{\alpha }\;\sin\beta +\Delta \theta_{y4}\;R\;\ddot{\alpha }\;\sin\beta -\Delta \theta_{y3}\;R\;{\dot{\alpha }}^{2}\;\cos\beta -\\ & \;\Delta \theta_{y4}\;R\;{\dot{\alpha }}^{2}\;\cos\beta -\Delta \theta_{x3}\;R\;{\dot{\alpha }}^{2}\;\sin\beta -\Delta \theta_{x4}\;R\;{\dot{\alpha }}^{2}\;\sin\beta +\\ & \;2R\;\dot{\alpha }\;\omega_{\mathrm{ie}}\;\cos L\;\sin\alpha -2\Delta \theta_{y4}\;R\;\dot{\alpha }\;\omega_{\mathrm{ie}}\;\sin L\;\cos\beta -\\ & \;2\Delta \theta_{x4}\;R\;\dot{\alpha }\;\omega_{\mathrm{ie}}\;\sin L\;\sin\beta \end{array}$$

### 4.3. Calibration Method Design

**Figure 11:**
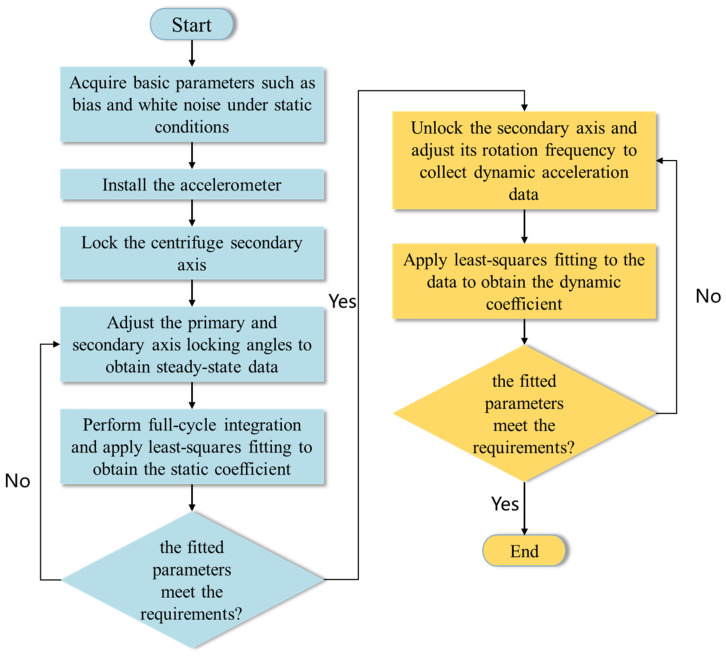
Flowchart of the dynamic calibration procedure.

Building upon the analysis of error sources and specific force inputs discussed in Section 2, a calibration method is proposed to characterize the dynamic performance of the accelerometer. In this approach, full-cycle integration is employed to mitigate the influence of periodic and systematic errors. As shown in Figure 11, the method primarily involves determining the accelerometer’s scale factor and its dynamic compensation coefficient.

#### Error Calibration Model Development

Assuming constant angular velocities for the primary axis Ω and the secondary axis ω (both constants), the calibration model is obtained by substituting Equation (37) into the dynamic error model (16). In the computation process, the specific force on the input axis is expanded to first-order terms, with higher-order small quantities neglected. Given that $K_{a}$ is a small quantity, only the nominal value of $\dot{a}$ is retained in calibration computations. Consequently, the following error calibration model is established for the quartz flexure accelerometer:

(40) $$\begin{array}{cc} E & =K_{1}\;(K_{0}-\Delta \theta_{y3}\;g-\Delta \theta_{y4}\;g-\Delta x\;{\dot{\alpha }}^{2}-\Delta R\;{\dot{\alpha }}^{2}\;\cos\beta -R\;{\dot{\alpha }}^{2}\;\cos\beta -\\ & \;\Delta \theta_{y2}\;g\;\cos\beta +\Delta \theta_{x2}\;g\;\sin\beta +\Delta \theta_{z3}\;R\;{\dot{\alpha }}^{2}\;\sin\beta +\Delta \theta_{z4}\;R\;{\dot{\alpha }}^{2}\;\sin\beta -\\ & \;\Delta \theta_{y0}\;g\;\cos\alpha \;\cos\beta -\Delta \theta_{y1}(\alpha )\;g\;\cos\alpha \;\cos\beta +\Delta \theta_{x0}\;g\;\cos\alpha \;\sin\beta +\\ & \;\Delta \theta_{x0}\;g\;\cos\beta \;\sin\alpha +\Delta \theta_{x1}(\alpha )\;g\;\cos\alpha \;\sin\beta +\Delta \theta_{x1}(\alpha )\;g\;\cos\beta \;\sin\alpha +\\ & \;\Delta \theta_{y0}\;g\;\sin\alpha \;\sin\beta +\Delta \theta_{y1}(\alpha )\;g\;\sin\alpha \;\sin\beta -2\;R\;\dot{\alpha }\;\omega_{\mathrm{ie}}\;\sin L\;\cos\beta -\\ & \;2\;\Delta \theta_{y4}\;R\;\dot{\alpha }\;\omega_{\mathrm{ie}}\;\cos L\;\sin\alpha +2\;\Delta \theta_{z4}\;R\;\dot{\alpha }\;\omega_{\mathrm{ie}}\;\sin L\;\sin\beta +\varepsilon )+\\ & \;K_{a}[(R\;\sin\beta )\;{\dot{\alpha }}^{2}\;\beta +2R\;\omega_{\mathrm{ie}}\;\dot{\alpha }\;\dot{\beta }\;\sin L\;\sin\beta ] \end{array}$$

The azimuth axis is locked to facilitate calibration of the scale factor $K_{1}$. Since $K_{0}$ and the noise term ε are known, only $K_{1}$ remains to be calibrated. By eliminating the influence of $K_{0}$ and ε, the Equation simplifies to the following:

(41) $$\begin{array}{cc} E & =K_{1}\;(-\Delta \theta_{y3}\;g-\Delta \theta_{y4}\;g-\Delta x\;{\dot{\alpha }}^{2}-\Delta R\;{\dot{\alpha }}^{2}\;\cos\beta -R\;{\dot{\alpha }}^{2}\;\cos\beta -\Delta \theta_{y2}\;g\;\cos\beta +\\ & \;\Delta \theta_{x2}\;g\;\sin\beta +\Delta \theta_{z3}\;R\;{\dot{\alpha }}^{2}\;\sin\beta +\Delta \theta_{z4}\;R\;{\dot{\alpha }}^{2}\;\sin\beta -\Delta \theta_{y0}\;g\;\cos\alpha \;\cos\beta -\\ & \;\Delta \theta_{y1}(\alpha )\;g\;\cos\alpha \;\cos\beta +\Delta \theta_{x0}\;g\;\cos\alpha \;\sin\beta +\Delta \theta_{x0}\;g\;\cos\beta \;\sin\alpha +\\ & \;\Delta \theta_{x1}(\alpha )\;g\;\cos\alpha \;\sin\beta +\Delta \theta_{x1}(\alpha )\;g\;\cos\beta \;\sin\alpha +\Delta \theta_{y0}\;g\;\sin\alpha \;\sin\beta +\\ & \;\Delta \theta_{y1}(\alpha )\;g\;\sin\alpha \;\sin\beta -2R\;\dot{\alpha }\;\omega_{\mathrm{ie}}\;\sin L\;\cos\beta ) \end{array}$$

A full-cycle integration is performed on the expression to suppress periodic error terms, resulting in the following relation:

(42) $$\begin{array}{cc} \int_{0}^{KT}E & =\int_{0}^{KT}K_{1}\;a_{i}\\ & =\int_{0}^{KT}K_{1}(-\Delta \theta_{y3}\;g-\Delta \theta_{y4}\;g-\Delta x\;{\dot{\alpha }}^{2}+\Delta \theta_{x2}\;g\;\sin\beta +\Delta \theta_{z3}\;R\;{\dot{\alpha }}^{2}\;\sin\beta +\\ & \;\Delta \theta_{z4}\;R\;{\dot{\alpha }}^{2}\;\sin\beta -2\;R\;\dot{\alpha }\;\omega_{\mathrm{ie}}\;\sin L\;\cos\beta )dt \end{array}$$

where: $T=\frac{2\pi }{\omega }\;K\in N^{*}$.

Here, $a_{m}$ depends on β, which is varied using the secondary axis. Twelve equally spaced angles should be selected as:

$$\beta =2\pi \left(i-1\right)/12$$

where: i=1,2,…,12.

This should be reformulated as:

(43) $$E\left(\beta \right)=K_{1}\cdot f_{K_{1}}\left(\beta \right)+\varepsilon \left(\beta \right)$$

The observation vector should be defined as:

(44) $$E={\left[\begin{array}{c} E(\beta_{1})\\ E(\beta_{2})\\ \vdots \\ E(\beta_{12}) \end{array}\right]}_{12\times 1}$$

The design matrix Φ is defined as:

(45) $$\Phi ={\left[\begin{array}{c} f_{K_{1}}(\beta_{1})\\ f_{K_{1}}(\beta_{2})\\ \vdots \\ f_{K_{1}}(\beta_{12}) \end{array}\right]}_{12\times 1}$$

The error model is expressed as:

(46) $$E=\Phi K_{1}+\varepsilon$$

(47) $$J(K_{1})=\|E-\Phi K_{1}{\|}^{2}$$

The least-squares solution matrix is obtained as:

(48) $${\hat{K}}_{1}={\left(\Phi^{T}\;\Phi \right)}^{-1}\Phi^{T}\;E$$

Based on ${\hat{K}}_{1}$, a test calibration for ${\hat{K}}_{a}$ should be performed.

The secondary axis should be unlocked and its frequency varied to collect dynamic data. Ten different frequencies should be selected, and 20 data points should be sampled uniformly within 0.05*T* to 1*T* at each frequency. The collected data are fit using least squares.

The resulting least squares matrix is:

(49) $${\hat{K}}_{a}={\left(\Phi^{T}\Phi \right)}^{-1}\Phi^{T}E$$

The observation vector is defined as:

(50) $$E={\left[\begin{array}{c} E(\beta_{1},\omega_{1})\\ E(\beta_{2},\omega_{1})\\ \vdots \\ E(\beta_{20},\omega_{1})\\ E(\beta_{1},\omega_{2})\\ \vdots \\ E(\beta_{20},\omega_{10}) \end{array}\right]}_{200\times 1}$$

The design matrix Φ is defined as:

(51) $$\Phi ={\left[\begin{array}{c} f_{K_{a}}(\beta_{1},\omega_{1})\\ f_{K_{a}}(\beta_{2},\omega_{1})\\ \vdots \\ f_{K_{a}}(\beta_{20},\omega_{1})\\ f_{K_{a}}(\beta_{1},\omega_{2})\\ \vdots \\ f_{K_{a}}(\beta_{20},\omega_{10}) \end{array}\right]}_{200\times 1}$$

The specific procedure for dynamic calibration of the accelerometer is outlined as follows:

Step 1: Obtain baseline parameters such as bias and white noise interference under static conditions using gravity-based methods.

Step 2: Install the accelerometer on a dual-axis precision centrifuge and adjust the installation angle as shown in Figure 9.

Step 3: Begin testing the accelerometer scale factor. Lock the secondary axis and set the primary axis angular velocity Ω.

Step 4: Vary the locking angle of the secondary axis to produce distinct acceleration inputs, selecting 12 evenly spaced angles from 0 to 2π. For each angle, collect accelerometer responses within 0.004T to 1T (T being the primary axis period) to mitigate initial transient effects.

Step 5: Remove calculable or measurable errors (e.g., Coriolis acceleration), conduct a full-cycle integration (the data collected are presented in Table 3), and perform least-squares fitting to estimate the scale factor.

Step 6: Calculate fitting evaluation metrics (e.g., mean squared error and coefficient of determination) to assess whether test results meet performance criteria.

Step 7: Unlock the secondary axis and incrementally adjust its rotational speed from 10 to 100 rad/s to capture system responses at each frequency.

Step 8: Eliminate measurable errors (e.g., Coriolis error) from the data. Using the scale factor obtained in Step 5, apply least-squares fitting to determine the dynamic coefficient (the data collected are presented in Table 4).

Step 9: Evaluate the fitting results using indicators such as MSE and R-squared to confirm whether the test criteria are fulfilled.

## 5. Simulation Verification and Evaluation

A simulation model was developed to validate the calibration procedure. The simulation assumes a test location at 45.0000° latitude, with dual-axis centrifuge errors in the order of $10^{-5}$ [38,46]. The primary axis rotation speed is set to 8.0829 rad/s, corresponding to an input acceleration of 10 g. A nominal scale factor of $K_{1}=1.2$ and a theoretical dynamic coefficient of $K_{a}=-5.5180430\times 10^{-4}$ are used in the simulation. Simulation results demonstrate that the proposed method achieves high calibration accuracy for both the scale factor and dynamic coefficient. Monte Carlo simulations further validate the method’s robustness under large-sample conditions by quantifying the associated uncertainty.

### 5.1. Single Static Test

The lock angle of the secondary axis is varied to collect test data, followed by full-cycle integration. Representative processed data, excluding Coriolis acceleration, are presented in Table 3.

A least-squares fit of the data yields the following static model parameters:

Scale factor: ${\hat{K}}_{1}\approx 1.200947$;
Mean squared error: Mse≈0.011670;
Root mean squared error: RMse≈0.108026;
Coefficient of determination: $R^{2}\approx 0.999996$;
95% confidence interval: [1.199332, 1.202562].

**Figure 12:**
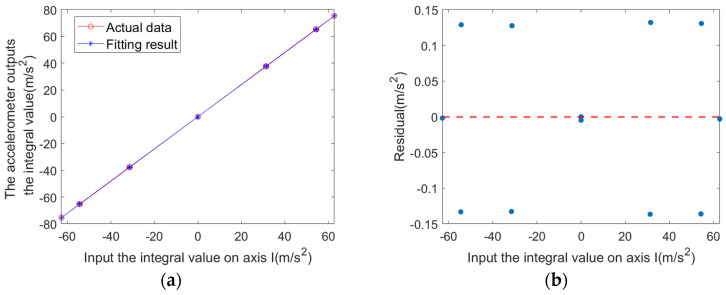
Static test results: (**a**) measured data and fitting curve; (**b**) residual plot.

A comparison of the fitted results with the simulated data is shown in Figure 12a, and the corresponding residuals are plotted in Figure 12b.

### 5.2. Single Dynamic Test

The primary and secondary axes are rotated simultaneously to collect dynamic test data. A portion of the data is shown in the table below. A portion of the processed data (with Coriolis acceleration removed) is shown in Table 4. A least-squares fitting is performed on the data to estimate the dynamic model parameter:

Dynamic factor: ${\hat{K}}_{a}\approx -5.5119358\times 10^{-4}$;
(theoretical value is $-5.5180430\times 10^{-4}$);
Mean squared error: Mse≈0.0142028;
Root mean squared error: RMse≈0.1191753;
Coefficient of determination: $R^{2}\approx 0.9999956$;
95% confidence interval: [−0.0005553, −0.0005471].

**Figure 13:**
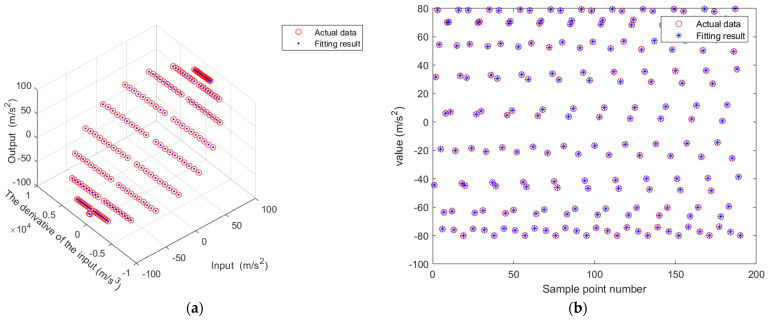
Dynamic test results: (**a**) comparison between actual data and fitting result; (**b**) comparison diagram of actual data and fitting results (2D).

**Figure 14:**
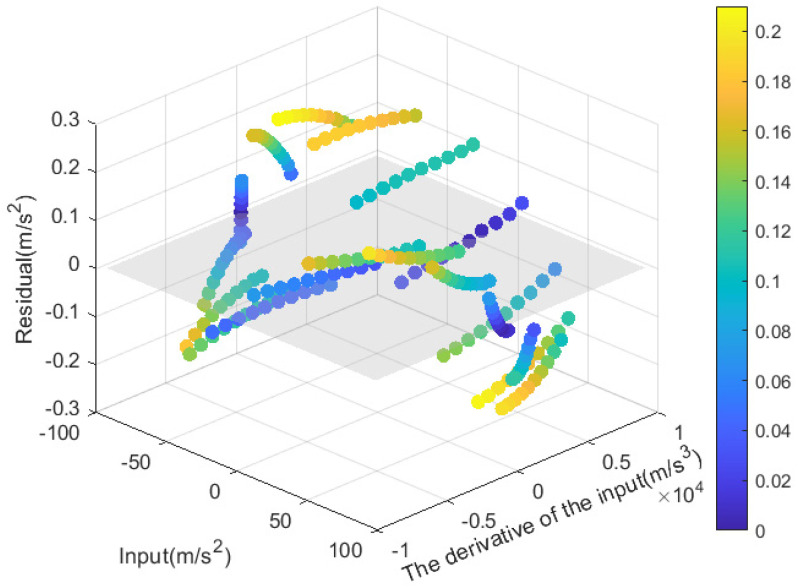
Dynamic test residual plot.

The comparison between the fitted results and the actual output is shown in Figure 13a,b, while the residuals are plotted in Figure 14.

### 5.3. Monte Carlo Simulation-Based Analysis of Accelerometer Coefficient Uncertainty

Although analytical uncertainty expressions for the static and dynamic coefficients can be derived from their mathematical formulations, they are often too complex for practical application. Moreover, the structural complexity of these expressions hampers the direct evaluation of how individual factors contribute to overall uncertainty. To address this, the Monte Carlo method is employed to conduct extensive sampling based on the statistical properties of each term, enabling insight into the resulting uncertainty distribution [47].

The number of Monte Carlo simulations for both static and dynamic tests is 1200 runs each.

**Figure 15:**
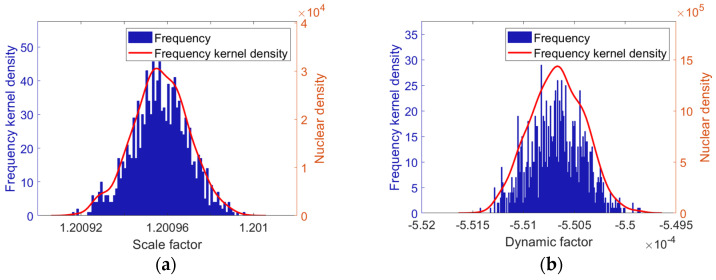
Monte Carlo simulation results: (**a**) histogram of scale factor frequency distribution; (**b**) histogram of dynamic coefficient frequency distribution.

The Monte Carlo distribution for the static test is illustrated in Figure 15a, where $K_{1}$ exhibits characteristics of an approximately normal distribution, with a mean of 1.20096 and a standard deviation of $1.3\times 10^{-5}$, yielding a relative standard deviation of 0.0011%. The variation of all measured values is confined within $\pm 4\times 10^{-5}$ relative to the mean value. To characterize the probability density distribution of the samples $K_{1_{i}}$, we employ kernel density estimation (KDE). The estimated value of the KDE at point x is given by the following:

(52) $${\hat{f}}_{h}(x)=\frac{1}{nh}\sum_{i=1}^{n}K\left(\frac{x-K_{1_{i}}}{h}\right)$$

where K(·) denotes the kernel function (Gaussian kernel is used), and h is the smoothing bandwidth (set to $3.256\times 10^{-6}$). The resulting red curve is shown in Figure 15a.

In large-sample scenarios, the proposed static calibration method is capable of accurately identifying the scale factor of the quartz flexure accelerometer. The calibration accuracy achieved for the scale factor is comparable to that of current mainstream methods [46,48].

As shown in Figure 15b, the Monte Carlo distribution of the parameter $K_{a}$ for dynamic testing approximately follows a normal distribution, with a mean of $-5.5067\times 10^{-4}$ and a standard deviation of $2.6\times 10^{-7}$, yielding a relative standard deviation of −0.048%. The variation of all measured values is confined within $\pm 1\times 10^{-6}$ relative to the mean value. Based on Equation (52), the kernel density curve is plotted with $h=7.24\times 10^{8}$, as shown in Figure 15b.

In large-scale simulations, the proposed dynamic calibration approach is shown to accurately identify the dynamic coefficient of the quartz flexure accelerometer.

## 6. Conclusions

This study proposes a calibration model grounded in the operating principles of quartz flexure accelerometers, incorporating the dynamic characteristics of input acceleration. In addition, a novel relative dynamic error index is introduced to quantitatively evaluate calibration effectiveness with respect to the accelerometer’s dynamic response. A dynamic parameter calibration method is subsequently developed using a dual-axis precision centrifuge. The method’s effectiveness and calibration accuracy are verified through simulation studies.

Simulation and theoretical analyses demonstrated the following:

(a) The proposed dynamic calibration model effectively accounts for the time-varying characteristics of acceleration inputs and achieves higher accuracy than conventional static models.
(b) For quartz flexure accelerometers exhibiting typical damping behavior, calibration with the proposed model significantly reduces dynamic errors within the standard operating range. Complete error compensation is achieved under ramp inputs, while sinusoidal inputs yield notable improvements in frequency domain accuracy.
(c) The dual-axis centrifuge-based calibration method effectively estimates the dynamic model parameters, confirming both the scientific validity and practical feasibility of the proposed model. The calibration of the dynamic parameter yields a relative standard deviation of −0.048%.

Future work will focus on exploring error mitigation strategies during calibration to further improve accuracy, building upon the proposed methodology. An experimental platform will also be developed for the physical validation and performance evaluation of the proposed method, with particular attention paid to its robustness and effectiveness in the presence of environmental noise. In addition, whether the response characteristics of the accelerometer change significantly with different input signals, and whether the proposed calibration model can accommodate such variations, will be further validated through experiments on the physical platform.

## Figures and Tables

**Table 1:** Dynamic error values of static and dynamic models at various frequencies.

**Frequency** ω **(rad/s)**	**0.100**	**0.231**	**0.586**	**1.485**	**3.765**	**9.546**
Static model error $\varepsilon_{d}$	6.61 × 10^−5^	3.87 × 10^−4^	9.78 × 10^−4^	2.431 × 10^−3^	5.458 × 10^−3^	1.008 × 10^−2^
Dynamic model error $\varepsilon_{d}$	3.323 × 10^−6^	7.674 × 10^−6^	4.933 × 10^−6^	1.251 × 10^−4^	3.172 × 10^−4^	8.073 × 10^−4^
**Frequency** ω **(rad/s)**	**24.201**	**61.359**	**155.568**	**572.237**	**830.218**	**1000**
Static model error $\varepsilon_{d}$	2.832 × 10^−2^	7.253 × 10^−2^	1.880 × 10^−1^	2.797 × 10^−1^	4.192 × 10^−1^	5.126 × 10^−1^
Dynamic model error $\varepsilon_{d}$	2.086 × 10^−3^	5.908 × 10^−3^	2.382 × 10^−2^	4.910 × 10^−2^	1.135 × 10^−1^	1.781 × 10^−1^

**Table 2:** Coordinate system transformation relations.

Coordinate System	Error Term	Coordinate Transformation Matrix
Geographic reference frame $O_{0}X_{0}Y_{0}Z_{0}\;$		
Main axis sleeve reference frame $O_{1}X_{1}Y_{1}Z_{1}$	$\Delta \theta_{x0}\left(\alpha \right),\Delta \theta_{y0}\left(\alpha \right)$	$T_{1}^{0}=\mathrm{rot}(x_{0},\Delta \theta_{x0})\cdot \mathrm{rot}(y_{0},\Delta \theta_{y0})$
Main axis reference frame $O_{2}X_{2}Y_{2}Z_{2}\;$	$\begin{array}{l} \Delta x_{1}(\alpha ),\;\;\;\;\Delta y_{1}(\alpha ),\;\;\;\;\Delta z_{1}(\alpha )\\ \Delta \theta_{x1}(\alpha ),\;\;\;\;\Delta \theta_{y1}(\alpha ) \end{array}$	$T_{2}^{1}=\mathrm{tran}\left(\Delta x_{1}(\alpha ),\Delta y_{1}(\alpha ),\Delta z_{1}(\alpha )\right)\cdot \mathrm{rot}(x_{1},\Delta \theta_{x1}(\alpha ))\cdot \mathrm{rot}(y_{1},\Delta \theta_{y1}(\alpha ))\cdot \mathrm{rot}(z_{1},\alpha )$
Azimuth axis reference frame $O_{3}X_{3}Y_{3}Z_{3}\;$	$\Delta R,\Delta \theta_{x2},\Delta \theta_{y2}$	$T_{3}^{2}=\mathrm{tran}(R+\Delta R,0,h)\cdot \mathrm{rot}(x_{2},\Delta \theta_{x2})\cdot \mathrm{rot}(y_{2},\Delta \theta_{y2})\cdot \mathrm{rot}(z_{2},\beta )$
Accelerometer casing reference frame $O_{4}X_{4}Y_{4}Z_{4}\;$	$\Delta \theta_{x3},\Delta \theta_{y3},\Delta \theta_{z3},\Delta x,\Delta y,\Delta z$	$T_{4}^{3}=\mathrm{rot}(x_{3},\Delta \theta_{x3})\cdot \mathrm{rot}(y_{3},\Delta \theta_{y3})\cdot \mathrm{rot}(z_{3},\Delta \theta_{z3})\cdot \mathrm{tran}(\Delta x,\Delta y,\Delta z)$
Accelerometer pendulum assembly reference frame $O_{5}X_{5}Y_{5}Z_{5}\;$	$\Delta \theta_{x4},\Delta \theta_{y4},\Delta \theta_{z4}$	$T_{5}^{4}=\mathrm{rot}(x_{4},\Delta \theta_{x4})\cdot \mathrm{rot}(y_{4},\Delta \theta_{y4})\cdot \mathrm{rot}(z_{4},\Delta \theta_{z4})$

**Table 3:** Results of the static test results.

**Integral value of the I-axis input (m/s^2^)**	**−75.3898240**	**−65.2895028**	**−37.6949120**	**0**	**37.6949120**	**65.2895028**
Accelerometer measured output (m/s^2^)	−62.7768581	−54.4759719	−31.4982265	−0.0000477	31.4975864	54.4738495
**Integral value of the I-axis input (m/s^2^)**	**75.3898240**	**65.2895028**	**37.694912**	**0**	**−37.6949120**	**−65.2895028**
Accelerometer measured output (m/s^2^)	62.7727251	54.2516002	31.2738693	−0.0040528	−31.2814464	−54.2577276

**Table 4:** Results of the dynamic test results.

**Ideal input along the I-axis (m/s^2^)**	**−90.8328217**	**84.4223511**	**−75.7923064**	**65.2545809**	**−52.6749421**	**39.036774**
Derivative of ideal I-axis input (m/s^3^)	619.6807592	−1367.4047251	2350.8017103	−3512.5138427	4806.0646933	−5255.3148971
Actual accelerometer output (m/s^2^)	−76.132558500	70.9614870	−64.6024280	56.1267940	−46.6890485	35.2704721
**Ideal input along the I-axis (m/s^2^** **)**	**−23.673606**	**8.0139039**	**8.0563864**	**−23.5288197**		
Derivative of ideal I-axis input (m/s^3^)	6506.7094863	−7650.6523934	8612.7862156	−9318.9423288		
Actual accelerometer output (m/s^2^)	−23.4083424	10.8270773	1.9438143	−14.4080055		

## Data Availability

Data are available upon request from the authors.

## Appendix A

In Equation (6), $\sigma_{1}$ and $\sigma_{2}$ are as follows, respectively:

$$\begin{array}{l} \sigma_{1}=k^{2}\theta_{O}\left(0\right)\sigma_{6}+2k^{2}{\dot{\theta }}_{O}\left(0\right)I_{O}+k^{2}\mu t{\mathrm{me}}_{0}+\mu^{2}Lk_{I}m+\left(-k\mu Lb_{1}m\right)+L^{2}{k_{P}}^{2}m^{2}\theta_{O}\left(0\right)\sigma_{6}+\\ \left(-2kI_{O}Lk_{I}m\right)+2{\dot{\theta }}_{O}\left(0\right)I_{O}L^{2}{k_{P}}^{2}m^{2}+\mu 2{k_{P}}^{2}m^{2}\theta_{O}\left(0\right)+\left(-L^{2}b_{1}k_{P}m^{2}\sigma_{6}\right)+\\ \left(-kLb_{1}m\sigma_{6}\right)+\mu Lk_{I}m\sigma_{32}+\left(-\mu 2b_{1}k_{P}m^{2}\right)+\left(-2I_{O}L^{2}k_{I}k_{P}m^{2}\right)+2kLk_{P}m\theta_{O}\left(0\right)\sigma_{6}+\\ 4k{\dot{\theta }}_{O}\left(0\right)I_{O}Lk_{P}m+K^{2}\theta_{O}\left(0\right)\sigma_{6}+2k\mu Lk_{P}m\theta_{O}\left(0\right)+2{\dot{\theta }}_{O}\left(0\right)I_{O}K^{2}+\mu R^{2}K^{2}\theta_{O}\left(0\right)+\\ 2kK\theta_{O}\left(0\right)\sigma_{6}+4k{\dot{\theta }}_{O}\left(0\right)I_{O}K+2k\mu RK\theta_{O}\left(0\right)+\left(-KLb_{1}m\sigma_{6}\right)+\left(-\mu RKLb_{1}m\right)+\\ \left(-2I_{O}KLk_{I}m\right)+2KLk_{P}m\theta_{O}\left(0\right)\sigma_{6}+4{\dot{\theta }}_{O}\left(0\right)I_{O}KLk_{P}m+2\mu RKLk_{P}m\theta_{O}\left(0\right) \end{array}$$

$$\begin{array}{l} \sigma_{2}=2k^{2}{\dot{\theta }}_{O}\left(0\right)I_{O}+\left(-k^{2}\theta_{O}\left(0\right)\sigma_{6}\right)+k^{2}\mu t{\mathrm{me}}_{0}+\mu^{2}Lk_{I}m+\left(-k\mu Lb_{1}m\right)+\\ \left(-L^{2}{k_{P}}^{2}m^{2}\theta_{O}\left(0\right)\sigma_{6}\right)+\left(-2kI_{O}Lk_{I}m\right)+2{\dot{\theta }}_{O}\left(0\right)I_{O}L^{2}{k_{P}}^{2}m^{2}+\mu 2{k_{P}}^{2}m^{2}\theta_{O}\left(0\right)+\\ L^{2}b_{1}k_{P}m^{2}\sigma_{6}+kLb_{1}m\sigma_{6}+\left(-\mu Lk_{I}m\sigma_{6}\right)+\left(-\mu 2b_{1}k_{P}m^{2}\right)+\left(-2I_{O}L^{2}k_{I}k_{P}m^{2}\right)+\\ \left(-2kLk_{P}m\theta_{O}\left(0\right)\sigma_{6}\right)+4k{\dot{\theta }}_{O}\left(0\right)I_{O}Lk_{P}m+\left(-K^{2}\theta_{O}\left(0\right)\sigma_{6}\right)+2k\mu Lk_{P}m\theta_{O}\left(0\right)+\\ 2{\dot{\theta }}_{O}\left(0\right)I_{O}K^{2}+\mu R^{2}K^{2}\theta_{O}\left(0\right)+\left(-2kK\theta_{O}\left(0\right)\sigma_{6}\right)+4k{\dot{\theta }}_{O}\left(0\right)I_{O}K+2k\mu RK\theta_{O}\left(0\right)+\\ KLb_{1}m\sigma_{6}+\left(-\mu RKLb_{1}m\right)+\left(-2I_{O}KLk_{I}m\right)+\left(-2KLk_{P}m\theta_{O}\left(0\right)\sigma_{6}\right)+\\ 4{\dot{\theta }}_{O}\left(0\right)I_{O}KLk_{P}m+2\mu RKLk_{P}m\theta_{O}\left(0\right) \end{array}.$$

where: $\sigma_{6}=\sqrt{\mu^{2}-4kI_{O}-4I_{O}Lk_{P}m-4I_{O}K}$.
